# Interactive Analysis of Epidemic Situations Based on a Spatiotemporal Information Knowledge Graph of COVID-19

**DOI:** 10.1109/ACCESS.2020.3033997

**Published:** 2020-10-26

**Authors:** Bingchuan Jiang, Xiong You, Ke Li, Tingting Li, Xiaojun Zhou, Liheng Tan

**Affiliations:** Institute of Geospatial Information, PLA Strategic Support Force Information Engineering University Zhengzhou 450052 China

**Keywords:** COVID-19, geographic knowledge graph, spatiotemporal big data, visual analysis

## Abstract

In view of the lack of data association in spatiotemporal information analysis and the lack of spatiotemporal situation analysis in knowledge graphs, this article combines the semantic web of the geographic knowledge graph with the visual analysis model of spatial information and puts forward the comprehensive utilization of the related technologies of the geographic knowledge graph and big data visual analysis. Then, it realizes the situational analysis of COVID-19 (Coronavirus Disease 2019) and the exploration of patient relationships through interactive collaborative analysis. The main contributions of the paper are as follows. (1) Based on the characteristics of the geographic knowledge graph, a patient entity model and an entity relationship type and knowledge representation method are proposed, and a knowledge graph of the spatiotemporal information of COVID-19 is constructed. (2) To analyse the COVID-19 patients’ situations and explore their relationships, an analytical framework is designed. The framework, combining the semantic web of the geographic knowledge graph and the visual analysis model of geographic information, allows one to analyse the semantic web by using the node attribute similarity calculation, key stage mining, community prediction and other methods. (3)An efficient epidemic prevention and anti-epidemic method is proposed which is of referential significance. It is based on experiments and the collaborative analysis of the semantic web and spatial information, allowing for real-time situational understanding, the discovery of patients’ relationships, the analysis of the spatiotemporal distribution of patients, super spreader mining, key node analysis, and the prevention and control of high-risk groups.

## I. Introduction

During the COVID-19 (Coronavirus Disease 2019) outbreaks, related methods and spatiotemporal big data have been continuously used to develop differentiated prevention and control measures. For example, measures such as COVID-19 patients’ maps, the population flow and distribution of potential patients, the spatiotemporal tracking of case trajectories, the allocation of medical resources in epidemic areas and the differentiated control of the epidemic have been used in the areas where they may have social and economic impacts. Applications include the spatial transmission of the epidemic, the dynamic analysis of disease conditions and the allocation of emergency resources. In general, spatiotemporal big data is of great importance in the emergency stage of the epidemic prevention and control, but rather weak in real-time disease monitoring and the spatiotemporal prediction of the epidemic’s spread. Due to the limits of the data model, it is difficult to associate spatiotemporal big data with multifactorial data related to the impact of the epidemic. Single, single domain and single mode data analyses which are used to conduct analyses and make predictions through the adjustment, correlation and optimization of an algorithm’s components that focus on a single domain. Thus, there is a lack of comprehensive analysis of multiple domains and multiple modalities. Therefore, it is necessary to consider comprehensively analysing the association of large-scale heterogeneous data and using heterogeneous data fusion to expand the multimodal knowledge support of epidemic infection prediction. This expansion requires integrating multiple data factors such as spatial and temporal distributions, population migration, and patients’ relationships for collaborative analysis. This integration must be done to analyse the underlying causes of the outbreaks and accurately discover the spatiotemporal mechanism of the epidemic spread, which will provide scientific and technological knowledge that can be used to prevent similar events from happening again.

Knowledge graphs, a branch of artificial intelligence and the most important way to represent knowledge in the big data era, have been widely used in intelligent search, intelligent Question & Answering (Q & A) [1], [2] and knowledge recommendation [3], [4]. Faced with the epidemic situation, relevant scholars and professionals in the field of knowledge graph research have constructed the knowledge graph of medical resources based on medical data, encyclopaedias, news, announcements, etc. The graph provides technical support such as medical resource tracing, epidemic early warnings, etc. Constructing a patient knowledge graph can realize the functions of super spreader mining, key node discovery, prevention and control measures for high-risk groups, etc.

In addition, the core of a knowledge graph is a large-scale semantic web [5]. The advantage of a semantic web is that it can well establish the semantic relationships between entities and store them in a simple formalized format for convenient and fast retrieval and analysis. Knowledge graphs are stored in triples (or quads or quintuples) for the purpose of weight reduction and simplification, which facilitates the identification by computers and reduces the computing requirements [6]. The advantage of the spatiotemporal information analysis model is that it can make full use of spatial location information, visualize the spatial distribution, and discover the temporal and spatial patterns by using the geographic analysis model. It is difficult to visualize the spatial distribution and spread of an epidemic situation when only use a semantic web. Complicated relationship information such as the patient’s social network relationships cannot be described when only use the geographic spatiotemporal model. Therefore, the authors consider the combination of the semantic web and geographic information model to give full play to their advantages to grasp the spatiotemporal distribution situation and relationship links.

In view of the lack of data association in spatiotemporal information analysis and the lack of spatiotemporal situation analysis in knowledge graphs, this article combines the semantic web of the geographic knowledge graph with the visual analysis model of spatial information and puts forward the comprehensive utilization of related technologies of the geographic knowledge graph and big data visual analysis. Then, it realizes the situational analysis of COVID-19 and the exploration of patient relationships through interactive collaborative analysis.

The main contributions of the paper are as follows.

1) Based on the characteristics of the geographic knowlege gradph, a patient entity model and an entity relationship type and knowledge representation method are proposed, and a knowledge graph of the spatiotemporal information of COVID-19 is constructed.
2) To analyse the COVID-19 patients’ situations and explore their relationships, an analytical framework is designed. The framework, combining the semantic web of the geographic knowledge graph and the visual analysis model of geographic information, allows one to analyse the semantic web by using the node attribute similarity calculation, key stage mining, community prediction and other methods.
3) An efficient epidemic prevention and anti-epidemic method that has referential significance is put forward. Based on experiments and the collaborative analysis of the semantic web and spatial information, it has been proved that this method it allows for real-time situational understanding, the discovery of patients’ relationships, the analysis of the spatiotemporal distribution of patients, super spreader mining, key node analysis, and the prevention and control of high-risk groups.

## II. Related Work

### A. Analysis of Infectious Disease Prediction

In the medical field, infectious disease prediction analysis has ranged from model predictions to big data-driven research. Increasingly more attention has been paid to the application of artificial intelligence and big data in the prevention and prediction of infectious diseases.

The early dynamic model provided a basic method using qualitative analysis and quantitative analysis to predict infectious diseases [7], [8]. However, due to the small sample data and limited model factors considered, the output results were a qualitative prevention test and management mode reports. With the development and utilization of multi-type sensors, various kinds of epidemic related data with more types and more complete samples have been obtained. Many scholars turned to machine learning and deep learning to achieve infectious disease prediction [9]–[11]. However, the training model is highly dependent on the size and accuracy of the training data set. The setting of the training model parameters is often subjective. The epidemic prediction itself has a large number of independent variables, making it a complicated problem, which could be solved using the combination of a complex network system and a simulation method [12] that could simulate the epidemic spreading process. Complex network and agent simulation [13], [14] that considers individual autonomous behaviour and the interaction between individuals can reflect the impact of a dynamic social network and individual autonomous behaviour on epidemic transmission. The core of this method is simulation modelling, but it does not take into account the attributes of all individuals (such as patients), the relationships between individuals, and the spatial and temporal factors (such as behavioural patterns, geographical distribution, and environmental impacts) that affect individuals.

Spatialtemporal elements are used in the medical research of infectious diseases in three ways. The first one is using them to analyse the spatial-temporal distribution and epidemic situation of diseases based on a spatial-temporal geographical simulation [15]–[17]. Such methods can provide good spatial and temporal references for the spatial spread of the epidemic, the dynamic analysis of the epidemic, and the allocation of emergency resources, but it is difficult to analyse the underlying causes of the outbreaks and the development of the epidemic. The second one is where the elements act as spatiotemporal influencing factors of the epidemic spreading in the process of building a simulation model of medical infectious diseases, which takes into account the geographical space-time factors in the prediction [18]. Such methods consider social and economic indicators, meteorological factors, population density, road traffic, spatial patterns, personnel movement and other factors, which provide a more comprehensive perspective and entry point for the current monitoring of the epidemic situation. However, the number of influencing factors that a single model can consider is limited. In addition, the variable factors such as changes in time and space could be hard to model. The third way is using them in interactive data analysis driven by multi-dimensional data. Using spatial-temporal big data technology, such methods integrate more environmental elements into collaborative analysis. The data-driven big data analysis method provides a new idea for the prediction analysis and mining of infectious diseases, which makes up for the shortcomings of the prediction model that is weak at complex model building [19]–[21].

The data-driven spatiotemporal analysis method enhances the collaborative analysis ability of multi-source heterogeneous data, emphasizes the relevance of data, makes better use of the correlation of data, allows for the mutual verification of data cross domains and disciplines, and improves the accuracy and scientificity of epidemic prediction. By fully using time series data, the intermediate process of the epidemic spread can be accurately controlled [22], [23]. In addition, prediction grading on different levels and stages of the epidemic makes it more conducive to establishing epidemic prevention and control measures. The introduction of different types of related data results in problems such as how to establish association models between data and how to realize the collaborative analysis of large-scale multi-source heterogeneous data; furthermore, such large scale data samples make the model calculation a problem [24].

### B. Geographical Knowledge Graph

The knowledge graph is essentially a networked knowledge base that is linked by entities with attributes through relationships. In other words, a knowledge graph is a knowledge base with a directed graph structure, where the nodes of the graph represent entities or concepts, and the edges represent various semantic relationships between entities/concepts.

A geographic knowledge graph is the expansion of a knowledge graph using geography, and it is a structured geo-semantic knowledge base. By formally describing the concept, entity, and attribute and their relationships in the field of geography, the concept and entity are connected with each other, forming a network knowledge structure [25].

The core of a geographic knowledge graph is to construct a large-scale geographic knowledge semantic web, which is essentially a large-scale directed network graph. The core technology is geographic entity association model building, which allows one to build relationships between large amounts of multi-source heterogeneous epidemic situation data and to achieve the collaborative analysis of multiple sources and multiple elements. With the rapid development of artificial intelligence, big data and other emerging technologies, geographic knowledge graphs have become an effective way to realize the intelligent organization of geographic information, and it has been rapidly recognized by relevant experts in the field of geography. Studies have been done on the aspects of geographic entity extraction [26], [27], topology and orientation relationship extraction [28] and geographic knowledge graph storage [29]–[32].

Related technology research results on the construction of geographic knowledge graphs mainly include the construction framework of the geographic knowledge graph put forward by Lu *et al.* [33]; the analysis and interpretation of the key technology of the construction of the geographic knowledge graph for the intelligent application of a virtual geographic environment that was conducted by Jiang *et al.* [25]; the CrowdGeoKG constructed by Zhou and Chen [34], who used wikidata and human geography knowledge to strengthen the geographic entities of OpenStreetMap; and the formal representation model of geographic knowledge proposed by Wang *et al.* [35], which can deal with the spatial, temporal and dynamic characteristics of geographic knowledge. A geographic knowledge graph can provide a good reference for forming the knowledge graph of the spatial-temporal information of COVID-19. The modelling and representation of spatial-temporal features could be realized using a knowledge graph. The knowledge graph of the spatiotemporal information of COVID-19 should include spatial and temporal characteristics, geographic entities, and data types such as space-time object, social network and regional environment data.

A large-scale spatiotemporal knowledge graph needs to combine spatiotemporal data representation models (such as maps, spatiotemporal distributions, etc.) for collaborative analysis. Sun and Sarwat [36] proposed a universal geographic knowledge graph indexing framework, Riso-tree, to implement semantic web and geospatial indexing, and constructed a location-aware search and query based on the geographic knowledge of interactive maps [37]. Having combined the characteristics of trajectory data and the definition of a knowledge graph, Wu *et al.* [38] extracted the entities, relationships, and attributes of trajectory data and constructed the trajectory map, which supports basic queries, range queries, nearest neighbour queries, keyword queries and trajectory mode queries. Xiao *et al.* [39] proposed a rumour propagation dynamics model based on an evolutionary game and anti-rumour information, and the model can effectively describe the propagation of rumours and the dynamic change rule of the influence of anti-rumour information, and further proposed a group behavior model for rumor and anti-rumor [40]. In paper [41], Yunpeng Xiao *et al.* used user multidimensional attributes and evolutionary games combined with the traditional susceptible-infected-recovered (SIR) epidemic model, which was used to quantify the impacts of external and internal driving factors on group state transitions during hotspot propagation.

In the application of geographic knowledge graphs, geographic knowledge association queries based on knowledge graphs are widely used. Typical examples are Geo-Wiki [42] (a geo-semantics sharing network system) and KIDGS [43] (a geographical knowledge-informed digital gazetteer service). Lin and Chen [44] and You and Lin [45] stated that to conduct VGE knowledge engineering, it is necessary to realize the intelligent transformation, “data-information-knowledge -smart” and realize intelligent virtual geographic environment services. Robert [46] presented a tentative conceptual framework for managing practical geographic knowledge, and the geographic knowledge base (GKB) includes geographic objects, geographic structures, geographic relations, geographic rules, geographic ontology, a gazetteer, physico-mathematical models, and external knowledge. Jiang *et al.* [47] stated that combining a spatial-temporal data model and semantic web model is one of the main ways to implement an intelligent geographic information service, and they also realized intelligent Q & A and interaction with a virtual geographic environment by using a geographic knowledge graph. Liu *et al.* [48] realized the intelligent querying of Chinese Guqin masters’ related information based on a knowledge base and a geographic information model. Liu *et al.* [49] built an event representation model centred on events that combined spatiotemporal and semantic features, which could organize multi-source heterogeneous data using knowledge graph technologies to represent terrorist event relationships and attribute information.

The geographic knowledge graph is an improvement of the knowledge graph, which has been successfully applied in fact-based knowledge Q & A, geographic knowledge association searches and intelligent interaction. In the face of the epidemic, the constructed epidemic Knowledge Q & A system and patient relationship query system have played positive roles in the popularization of epidemic knowledge and in self-protection measures. However, due to the incomplete correlation data and inadequate correlation analysis methods, it is difficult to carry out in-depth analysis, which makes it a problem to closely follow and further predict the epidemic situation.

In summary, the related research on knowledge graphs can direct the construction of epidemic knowledge graphs in these two aspects.

1) The construction of an ontology model of geographical knowledge. Geographical ontology research started earlier, and has formed a relatively complete set of ontology systems and ontology model construction methods, which can provide ontology system guidance for the construction of the COVID-19 patients’ spatiotemporal information knowledge graph; however, it needs to be improved based on the characteristics of the medical field.
2) The geographic knowledge extraction and representation model. This model has certain referential significance for the extraction of different geographical knowledge, especially knowledge with temporal and spatial variables such as spatiotemporal information and geographic events. However, it requires further research regarding the formalized representation of the geographic spatial-temporal model about the epidemic’s spread in order to build a model or representation that presents both factual knowledge and epidemic changes.

## III. Construction of COVID-19 Patients’ Information Knowledge Graph

**FIGURE 1.**
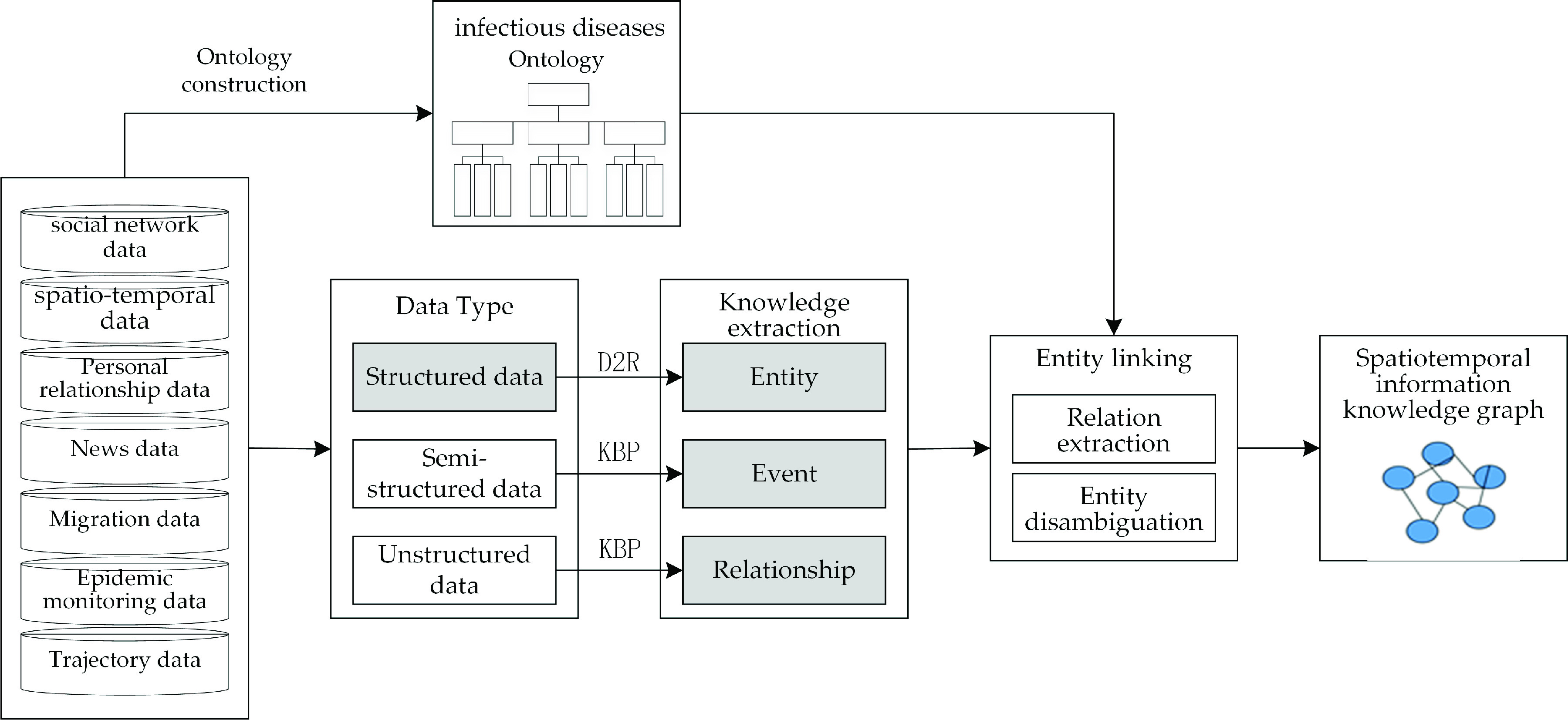
The basic framework of the spatial-temporal information knowledge graph of COVID-19 patients.

Referring to the basic process of building a geographic knowledge graph, the basic framework for building the spatial-temporal information knowledge graph of COVID-19 patients is shown in Figure 1. The knowledge sources of the spatiotemporal information knowledge graph of patients include the following: social network data, personal relationship data, news data, migration data, epidemic surveillance data, trajectory data, and basic geographic information data. The data sources, formats and extraction methods are shown in Table 1.

**TABLE 1:** Sources of the Spatiotemporal Information of COVID-19 Patients

Data category	Data Sources	Data Format	Extraction method	Knowledge type
Social network data	WeChat and Weibo data	Unstructured text	Wrapper extraction	Character entities, social relationships, geographic and hidden information
Personal relationship data	Household registration, WeChat, and Weibo	Structured household registration database, unstructured text	D2R, wrapper extraction	Character entities and character relationships
News data	News website	Unstructured text	Wrapper extraction	News
Migration data	Railway, civil aviation, and highway administrations	Structured database	Wrapper extraction	Tracking data and vehicle entities
Outbreak Monitoring data	National and provincial health committees	Unstructured text	Wrapper extraction	Patient entities and patient relationships
Trajectory data	Mobile positioning and surveys	Structured database	Wrapper extraction	Character entities and, displacement events
Basic geographic information data	Maps, Images, and Geographic Names	Structured database	Wrapper extraction	Geographical Name Entities and Geographical Knowledge

### A. Ontology Construction

The ontology layer of COVID-19 patients’ knowledge graph is a subset of geographic ontology.

#### 1) Define the Concept Set

ON_Concept:{ $C$} is defined as follows: { $C_{1}$: patients, $C_{2}$: residence, $C_{3}$: hospital, $C_{4}$: vehicle, $C_{5}$: location, $E$: event ontology}.

Here, $C_{1}$: refers to the confirmed COVID-19 patients, $C_{2}$: refers to the patients’ residence, $C_{3}$ refers to the hospital where the patients get medical treatment, $C_{4}$: refers to the patients’ means of transport, $C_{5}$: refers to the location of the patients; and E: event ontology, which mainly includes medical treatment events, confirmed events, travel events, contact events, shopping events, gathering events, home events and fever events.

Define the relationship in the ontology ON_Relationship as follows (take the patient ontology and other types of ontology as examples):

\begin{align*}&\hspace {-0.8pc}\{R\}=\{R(C_{1},C_{1}),R(C_{1},C_{2}),R(C_{1},C_{3}), \\&\qquad \qquad \qquad \qquad \qquad R(C_{1},C_{4}),R(C_{1},C_{5}),R(C_{1},E)\}\end{align*}

where,

①
$R(C_{1},C_{1})$ is the relationship between patients;
②
$R(C_{1},C_{2})$ is the relationship between the patients and the places they live, i.e., “residence”;
③
$R(C_{1},C_{3})$ is the relationship between patients and the hospitals where they get medical treatment, i.e., “hospital in”;
④
$R(C_{1},C_{4})$ is the relationship between patients and the transportation, i.e., “take”;
⑤
$R(C_{1},C_{5})$ is the relationship between the patients and their locations, i.e., “location”; and
⑥
$R(C_{1},E)$ is the relationship between patients and events, i.e., “has-event”.

#### 2) Entity Semantic Model and Relationship Definition

The entity conceptual model is shown in Table 2.

**TABLE 2:** Entity Conceptual Model

**FIGURE 2.**
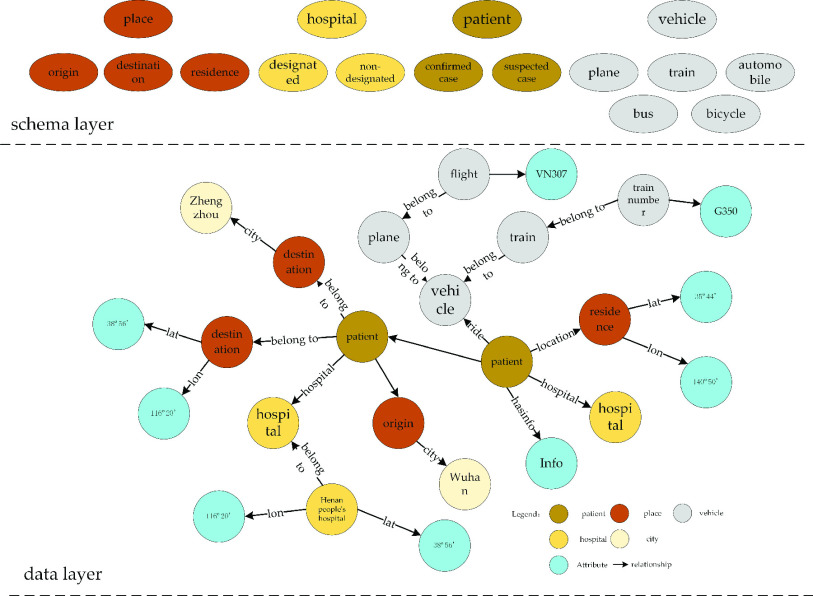
The COVID-19 patients’ entities and relationships.

Patient entity
+Patient entity identification code:Entity_ID
+Patient entity name:Name
+Patient entity classification:Classification
+Patient entity relationship:Relationship
+Patient entity properties:
+Patient entity location:
+Patient events:

The knowledge graph of COVID-19 patients has clear characteristic spatiotemporal information, mainly including the following, as shown in Figure 2.

① Physical position characteristics. All entities e have basic location characteristics, including $f_{e}$ = {lng,lat, $D$}, where lng represents longitude, lat represents latitude, and $D$ describes the scope of the entity and mainly refers to the administrative radius of the area where the patient is located.
② Triple related to the location. In the triple $(h,r,t)$, at least one of the two entities, $h$ and $t$, contains a location feature. At present, the entities with location features in the graph include residences, departures, destinations, hospitals for medical treatment, locations and so on.

#### 3) Event Semantic Model and Relationship Definition

An event includes 6 elements: who, when, where, action, state and what. An event, expressed as e, could be defined as a sixtuple as follows:

\begin{equation*}e=(E,T,P,A,S,W)\end{equation*}

The letters $E,T,P,A,S$ and $W$ represent the subject (including entities such as people and medical organizations), time, place, actions (such as trigger words for actions), state set and situations (event descriptions), respectively. For example, in “patient 23 returned to Taikang County by taking private car from Wuhan on January 7‘”, the event subject is “patients 23”, the time is “January 7”, the position is “Wuhan”, “Taikang county”, the actions are “take, return”, the state set is “return”, and the event description is “take private car to return”.

The relationships between events include constituent relationships, sequential relationships, causal relationships and correlation relationships. The relationship between events can be linked through time, place and participants.

For example: “Patient XXX, female, 25 years old, now living in the Evergrande Oasis of Minghu Office of the Economic Development Zone, contacted the returnees from Wuhan in Zhengzhou on January 18. She took the G658 high-speed rail (carriage 11) to Xinxiang on January 19 and went back to Zhengzhou on January 21 by high-speed rail G1813 (carriage 4). Then, she drove to Ruzhou, PingDingshan on the same day and drove back to Zhengzhou on January 30 at last. Since then, she had been stayed at home until fever symptoms occurred on February 1 when she drove to Zhengzhou Seventh People’s hospital. On February 3, she drove to the people’s Hospital of Henan Province for treatment and was diagnosed on February 6 ”

**FIGURE 3.**
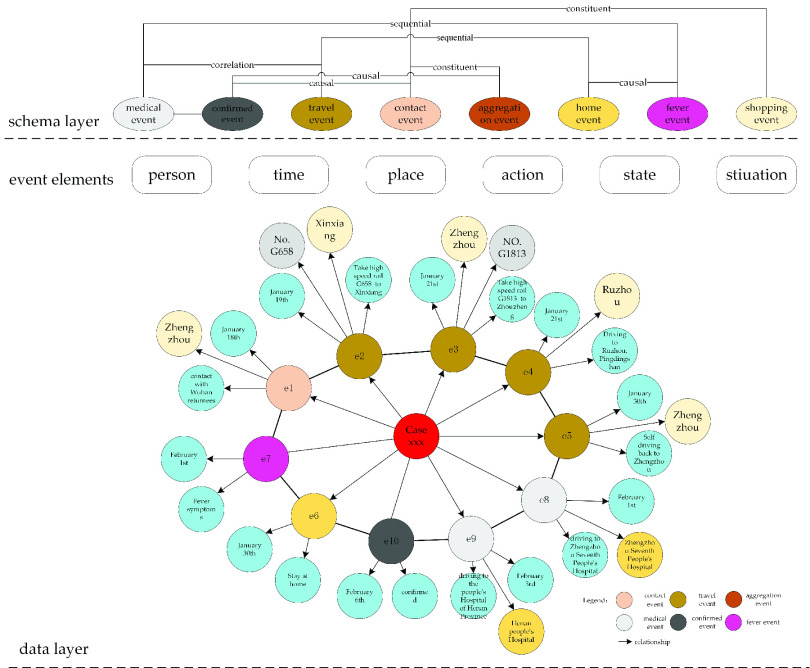
The schema of events and relationships.

As shown in Figure 3, there is a causal relationship between the contact event $e_{1}$ “contact with Wuhan returnees on January 18” and the fever event $e_{7}$ “fever symptoms on February 1”. The migration event $e_{2}$ “take G658 high-speed rail (carriage 11) to Xinxiang on January 19” and the migration event $e_{3}$ “take high-speed rail G1813 (carriage 4) to Zhengzhou on January 21” are sequential. The fever event $e_{7}$ “fever symptoms on February 1” and the event $e_{8}$ “drive to Zhengzhou Seventh People’s Hospital on February 1 ” have a causal relationship.

#### 4) Semantic Web Representation

**FIGURE 4.**
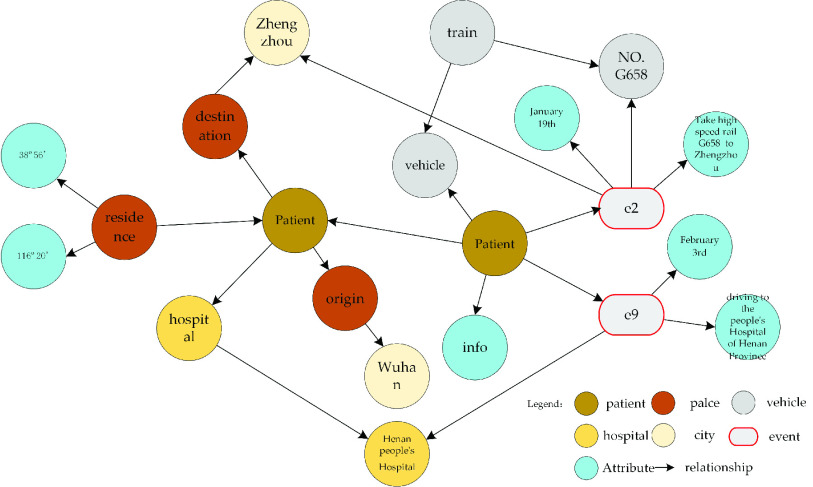
The representation of the COVID-19 spatiotemporal information knowledge graph.

The spatiotemporal information knowledge graph of COVID-19 patients expresses the concept, entity, attribute and event of the COVID-19 subfield with resource the description framework (RDF) as a triple, namely, “< s (subject), P (predicate), O (object) >”. Then, we establish the relationships among its elements, entities and events. It is represented by the directed graph of “point—edge”, as shown in Figure 4.

## IV. Analysis Method of Epidemic Situation Based on Spatiotemporal Information Knowledge Graph of COVID-19 Patients

### A. Analysis Tasks

To realize the monitoring and analysis of epidemic situations by combining the patient semantic relation network model and spatiotemporal information visual analysis model, the following analysis tasks need to be done:

1) Epidemic situation analysis: analyse the regional distribution of patients to understand the current situation,
2) Patients’ relationship analysis: analyse patients who are infected by gathering for super spreader mining,
3) Analysis of the spatial and temporal patterns of the patients: analyse patients’ activity areas to set up early warning and protection areas, and
4) Early warning and prediction of high-risk groups: find the possible cross-infection patients based on the relationships between patients and other entities.

### B. Analysis Framework

**FIGURE 5.**
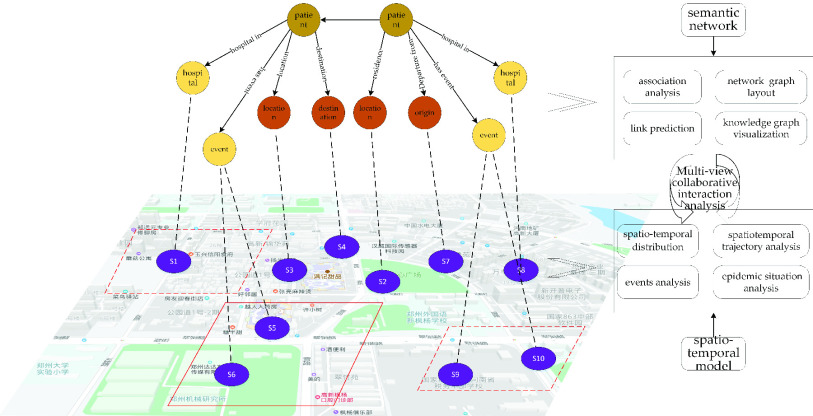
Collaborative analysis of the semantic web and spatiotemporal model.

As shown in Figure 5, the basic analytical framework for the comprehensive geographic knowledge graph is based on the knowledge graph semantic web model and the temporal and spatial information visual analysis model. Almost all nodes in the spatiotemporal information knowledge graph have spatiotemporal position information, and all types of nodes can be mapped to a unified spatiotemporal framework. This framework can take full advantage of the characteristics of the semantic web map analysis and geographical spatiotemporal analysis for comprehensive analysis and application. The graph analysis based on the knowledge graph semantic model includes functions such as relationship analysis, network graph distribution, link prediction, and graph visualization. The analysis based on spatiotemporal data models includes functions such as geographical spatiotemporal distribution, spatiotemporal trajectory analysis, character and event analysis, and spatiotemporal situation analysis.

### C. Node Clustering and Similarity Analysis of COVID-19 Patients’ Semantic Web

#### 1) Similarity Calculation of Patients’ Nodes Based on Node Attributes

The patients’ spatiotemporal information knowledge graph is highly dynamic. The entities in the network and the relationships between them would evolve over time and space. New nodes might show up while the existing ones might disappear, and currently disconnected nodes might be connected. Link prediction can be used to analyse the potential relationships between the confirmed patients and to report on an epidemic situation that requires an early warning.

Node similarity calculation methods are divided into attribute-based methods and link-based methods. The COVID-19 patients’ knowledge graph semantic web is a directed graph with relatively clear node attributes where the attribute-based method should be applied. It calculates the similarity by comparing the attribute values of the nodes. First, the attribute vectors are constructed for each node, and each attribute is regarded as a dimension of the multi-dimensional space. For example, $v_{p}$ and $v_{q}$ are two nodes in the network and both of them have m attributes, which could be expressed as follows:

\begin{align*}v_{p}=&(e_{p_{1}},e_{p_{2}},\cdots,e_{p_{m}})\\ v_{q}=&(e_{q_{1}},e_{q_{2}},\cdots,e_{q_{m}})\end{align*}

The nodes are mapped to the low dimensional space, and then the node similarity is calculated using the Euclidean distance formula and cosine similarity formula of vector space.

\begin{align*} \mathrm {dist}(v_{p},v_{q})=&\sqrt {\sum _{i=1}^{m}(v_{p_{i}}-v_{q_{i}})^{2}} \tag{1}\\ \mathrm {sim}(v_{p},v_{q})=&\frac {\vec v_{p}\cdot {\vec v_{q}}}{\|{v_{p}}\|\cdot \|{v_{q}}\|}\tag{2}\end{align*}

#### 2) Super Spreader Mining Based on Betweenness Centrality Algorithm

The betweenness centrality, which is the probability that a node is located on the shortest path connecting any two nodes, is closely related to the length of the path. The COVID-19 patients’ knowledge graph semantic web has rich knowledge of node attributes and relationships (edges), which can realize super spreader mining using the betweenness centrality algorithm.

The betweenness centrality of a node [50] refers to the ratio of the number of shortest paths passing through the node to the number of shortest paths between any two nodes in the network. Its value indicates the network’s control ability over information transmission. The larger the value is, the greater the number of shortest paths between any two nodes in the network passing through the node, and the more important the node.

Suppose that the number of shortest paths between node pair $j$ and $k$ is $g_{j,k}$, the number of shortest paths that pass through node $i$ is $g_{i,j}(i)$, and $C_{j,k}(i)$ represents the betweenness centrality of $i$, i.e., the probability that $i$ is on the shortest path between $j$ and $k$. The calculation formula for the betweenness centrality is as follows [51]:

\begin{equation*} \mathrm {BC}(i)=\sum \limits _{j\neq i\neq k\in v}\frac {g_{j,k}(i)}{g_{j,k}}\tag{3}\end{equation*}

$g_{j,k}(i)$ is the number of shortest paths between $j$ and $k$ that $i$ passes. $g_{j,k}$ is the number of the shortest paths between $j$ and $k$.

#### 3) Semantic Web Node Clustering Based on the Louvain Algorithm

COVID-19 patients’ spatiotemporal information knowledge graph is essentially a large-scale directed graph. The cluster analysis of network nodes is similar to the community detection of a social network. A community is a set of nodes that are similar to each other and different from other nodes in the network. The community detection algorithm helps to mine some nodes at the centre or edge of the community. For example, it can be used for the analysis of infected patients caused by a family gathering.

The Louvain algorithm [52] is a community detection algorithm based on Modularity, it is efficient in providing good results, and detecting hierarchical community structures. Its optimization goal is to maximize the Modularity of the community network.

The Modularity function is used to measure the quality of the results of the community detection algorithm. It can describe the compactness of detected communities [53]. Its function is defined as follows:

\begin{equation*} Q=\frac {1}{2m}\sum _{i,j}\left[{A_{i,j}-\frac {k_{i}k_{j}}{2m}}\right]\delta (c_{i},c_{j})\tag{4}\end{equation*}

where $m$ is the number of edges in the network, and $A$ is the adjacency matrix. If $c_{i}$ equals $c_{j}$, then $\delta (c_{i},c_{j})=1$; otherwise, it would be 0.

The formula can be further transformed into the following:

\begin{equation*} Q=\frac {1}{2m}\sum _{c}\left[{\sum \mathrm {in}-\frac {\left({\sum \mathrm {tot}}\right)^{2}}{2m}}\right]\tag{5}\end{equation*}

$\sum \mathrm {in}$ represents the sum of the weights of the edges in community $c$, and $\sum \mathrm {tot}$ represents the sum of the weights of the edges connected to nodes in community $c$.

The Louvain algorithm consists of the following steps.

Step 1: Traverse all the nodes $i$ in the network, assign a single node $i$ to the community of each neighbouring node, calculate the changes in the Modularity measure $Q$ represented by $\Delta Q$, and add it to the community of the neighbouring node with the maximum change in Modularity measure. Repeat this step until all nodes are no longer change, and the generated small community is the input of the second step.
Step 2: Compress the graph. All nodes in the same community are compressed into a new node. The weights of the edges between the nodes in the community are converted into the weights of the ring of the new node, and the edge weights between the communities are converted into the edge weights between the new nodes.
Step 3: Iterate these two steps until the algorithm is stable.

When node $i$ is assigned to the community of neighbouring node $j$, $\Delta Q$ can be calculated by formula 6

\begin{align*}&\hspace {-.5pc} \Delta Q=\left[{\frac {\sum \mathrm {in}+k_{i,in}}{2m}-\left({\frac {\sum \mathrm {tot}+k_{i}}{2m}}\right)^{2}}\right] \\&\qquad \qquad \qquad \qquad -\left[{\frac {\sum \mathrm {in}}{2m}-\left({\frac {\sum \mathrm {tot}}{2m}}\right)^{2}-\left({\frac {k_{i}}{2m}}\right)^{2}}\right]\tag{6}\end{align*}

The pseudocode of the Louvain algorithm is shown in Table 3. The time complexity of the Louvain community discovery algorithm is $\text{O}(n\mathrm {log}n)$, where $n$ is the number of nodes in the network. As the data scale increases, the time complexity of the algorithm increases by levels of $n\mathrm {log}n$.

**TABLE 3:** Pseudocode of Louvain Algorithm

Define directed node network graph structure as $G (V, E)$
1) Initialize each point in G as a community, calculate the module degree value at this time and store it in $Q_{1}, Q_{3} = Q_{1}$;
2) $Q_{2} = Q_{1}$;
3) for $i = 1$ to $n$ /* $n$ is the number of vertices in the network graph*/
4) if $d(v_{i}) == 1$ /* $d(x)$ calculate the degree of vertex */
5) add points to the community to which they are connected;
6) else
7) remove the vertex $v_{i}$ from the original community;
8) add $v_{i}$ to the community that makes $\Delta Q$ the largest;
9) end if
10)end for
11)calculate the modularity value at this time and store it in $Q_{1}$;
12) if $Q_{1} \unicode{0x003E} Q_{2}$, go to step 2;
13) end if;
14) merge the communities into one super point;
15) store the points contained in different hyper points into the corresponding collection array communities;
16) return communities

## V. Experiment

### A. Experiment Data

The experimental data is from Henan Province Government that have been collected from the confirmed COVID-19 patients in Henan Province in China (until March 13, 2020). After data cleaning, the patient knowledge graph of the spatiotemporal information is constructed with 2312 entities and 5055 entity relationships. The entity labels include “patient”, “train number”, “place of residence”, “place of departure”, “destination”, “hospital”, “event”, etc.

### B. Experimental Platform

**FIGURE 6.**
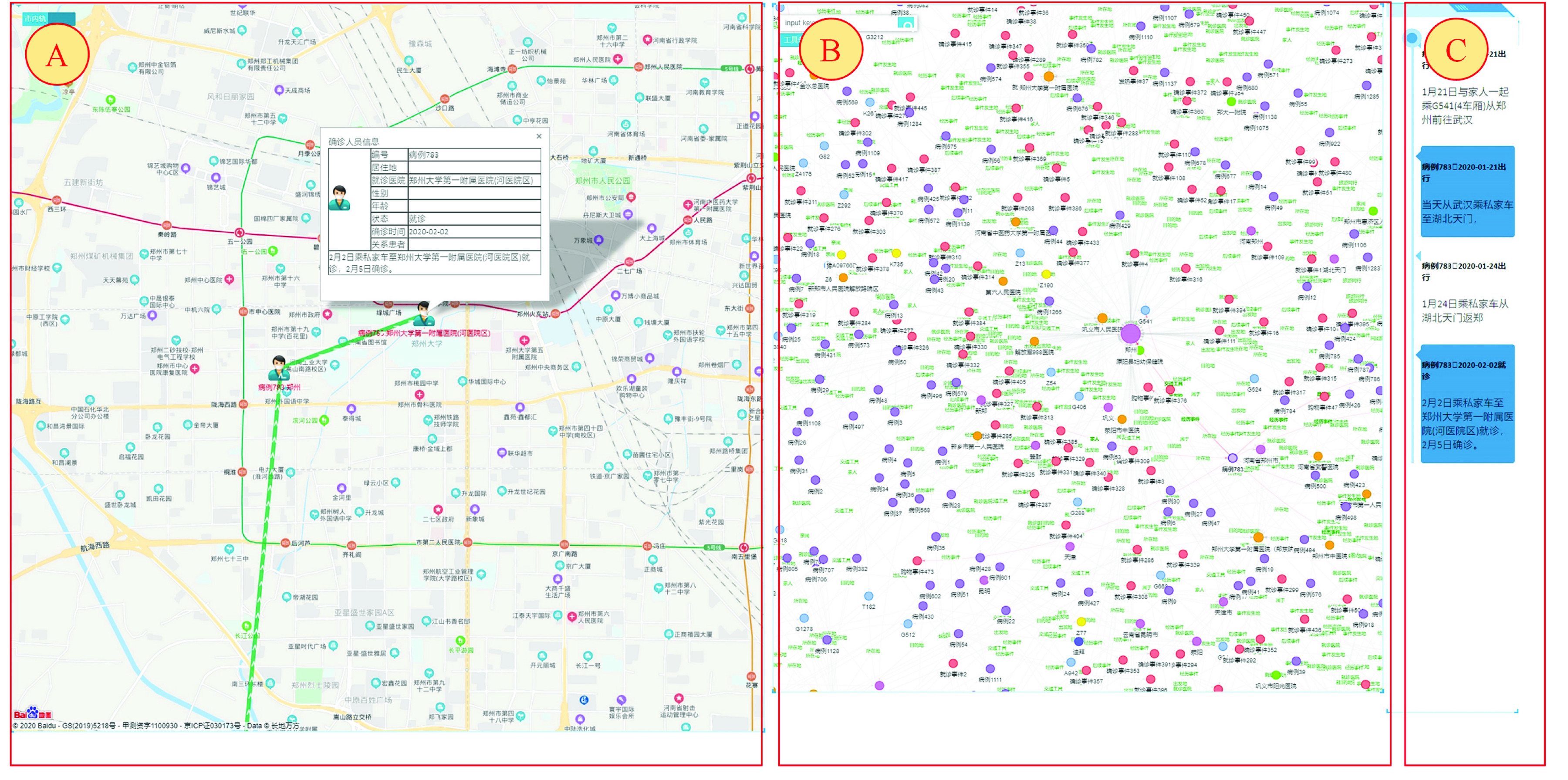
Visual analysis of the COVID-19 patient’s knowledge graph.

The experimental platform adopts the B/S architecture, which is developed and implemented based on the ECharts and Java languages. By taking advantage of both rich GIS information and the interactive visual analysis system, a visual analysis system of the spatiotemporal information knowledge graph of COVID-19 is constructed. The system interface is shown in Figure 6.

Figure 6 is the network analysis of the patients’ relationships in Henan Province, which uses the following components.

A: Maps and migration maps. It mainly displays the location information, urban trajectory and migration trajectory of patients.
B: Interactive visual analysis of the knowledge graph of COVID-19 patients. Through the collaborative analysis with the maps, the patient type analysis, the regional prevention and control situation analysis, the gathering infected patient analysis, high-risk group prevention and control, the city track analysis, and the missing reports and concealed cases analysis, the following functions are realized: patient relationship tracking, high-risk group prevention and control information release, event pattern analysis, etc.
C: Event axis. These lists are used to show the temporal and spatial characteristics of patients’ visiting events, diagnosis events, travel events, contact events, shopping events, gathering events, quarantine events and fever events.

### C. Knowledge Graph Semantic Web Analysis

#### 1) Analysis of Patients’ Types

**FIGURE 7.**
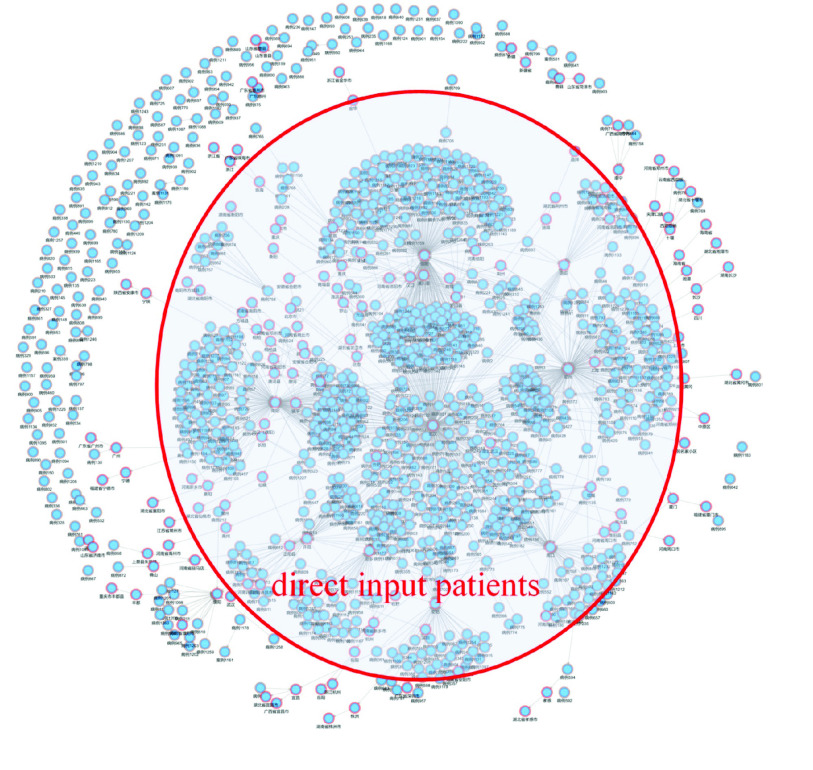
Analysis of patient types.

After screening the “patient” and “departure place” entities, in Figure 7, it can be clearly seen that in the early stage of the epidemic, most of the infected people are related to “Wuhan”, “Hankou” and other Hubei areas, belonging to the “direct input” patient type.

#### 2) Analysis of Regional Prevention and Control Situation

**FIGURE 8.**
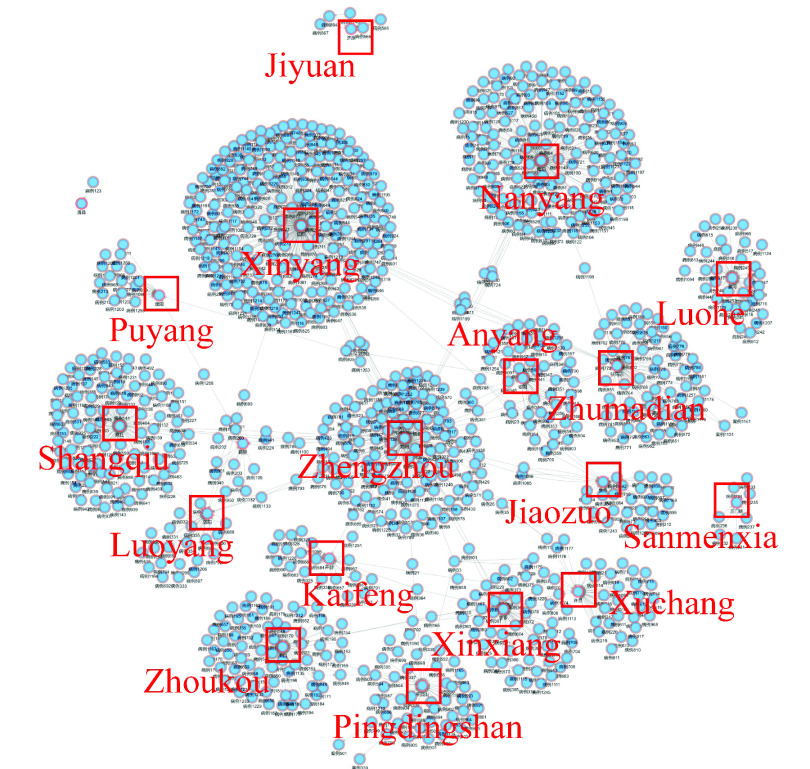
Analysis of regional prevention and control situation.

After screening the “patient” and “location” entities, as shown in Figure 8, it can be seen clearly that the patients are mainly located in “Zhengzhou”, “Xinyang”, “Pingdingshan”, “Nanyang”, “Jiaozuo”, “Shangqiu”, “Xinxiang”, “Kaifeng”, “Hebi”, “Luohe”, “Zhoukou”, etc. Some patients moved directly between different cities, resulting in cross-regional infections. There is no personnel movement in “Jiyuan”, “Luohe”, and “Sanmenxia” due to strict control measures.

#### 3) Patients Analysis of Family Gathering Infection

**FIGURE 9.**
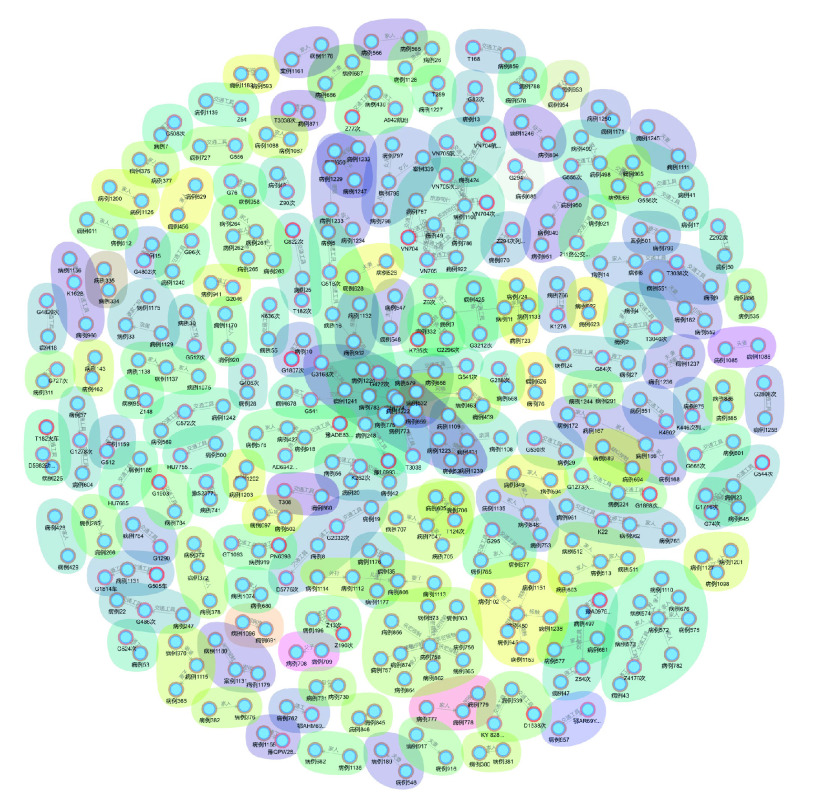
Patients’ analysis of family gathering infection.

After the character relationship is screened and the free node is removed, the high risk of infection from families has been proved by the Louvain group analysis, as shown in Figure 9.

Therefore, it is of necessity to implement quarantine measures for families.

#### 4) High-Risk Group Prevention and Control Analysis

**FIGURE 10.**
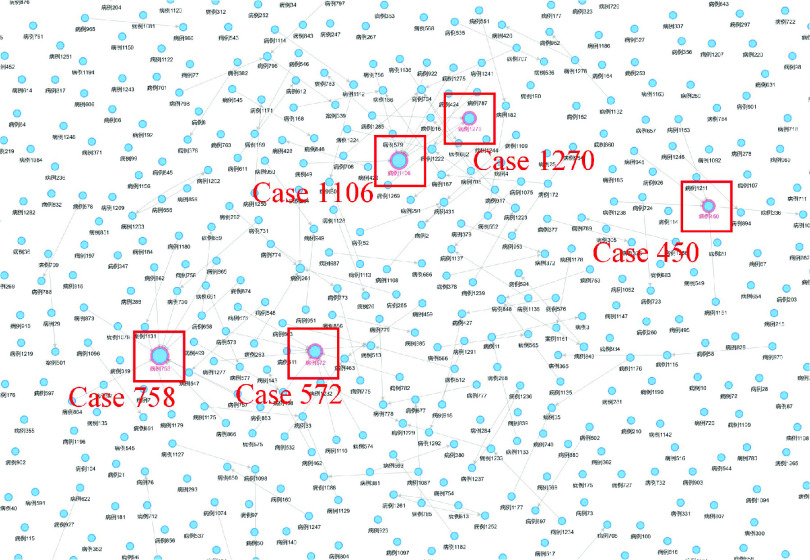
Analysis of super spreaders based on betweenness centrality.

**FIGURE 11.**
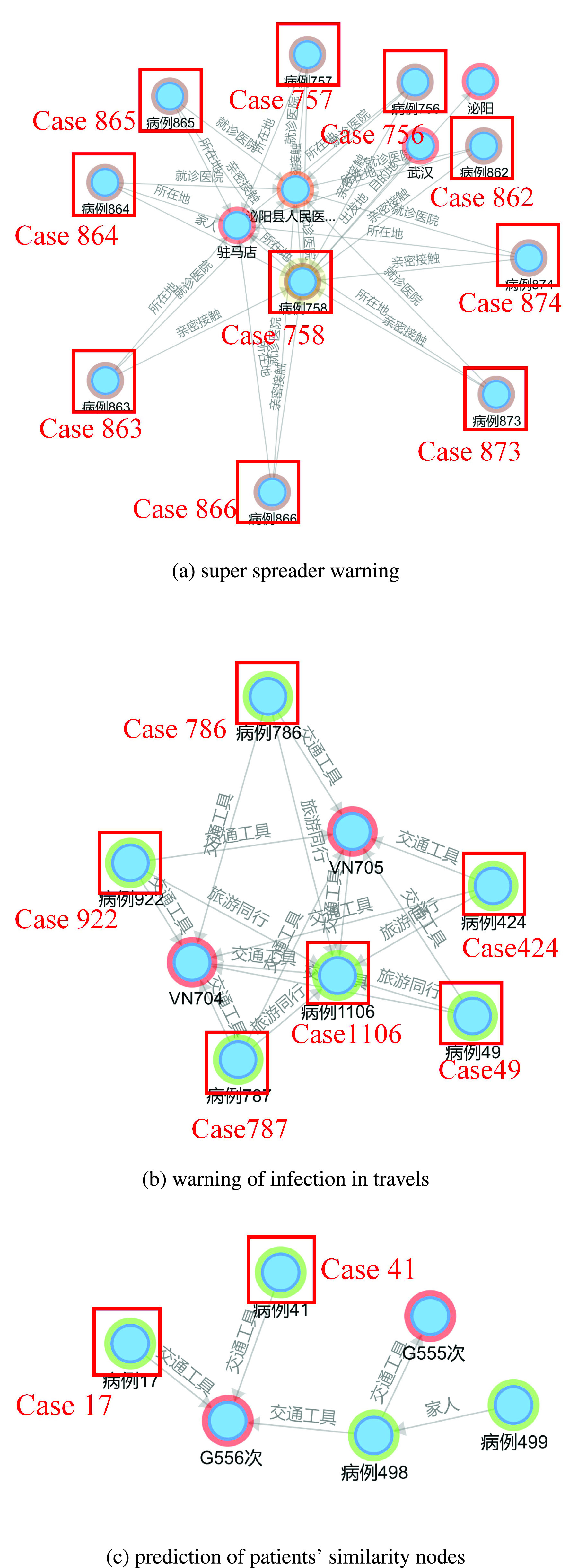
Analysis of typical cases.

There are several patients who are infected during family gatherings. For example, as shown in Figure 10, based on the betweenness centrality analysis, “case 450”, “case 572”, “case 758”, “case 1270” and “case 1106” all get infected by their family members they have contacted. These cases are typical super spreaders (as shown in Figure 11(a)), and thus it is necessary to remind the personnel who have close contact with them.

As shown in Figure 11(b), “Case 49”, “case 786”, “case 424”, “case 922” and “case 787” took the same flight to travel abroad on the same day. It proves the necessity of the release the key flight information in time for early warnings, and the passengers on this flight should be quarantined for particular observations.

**FIGURE 12.**
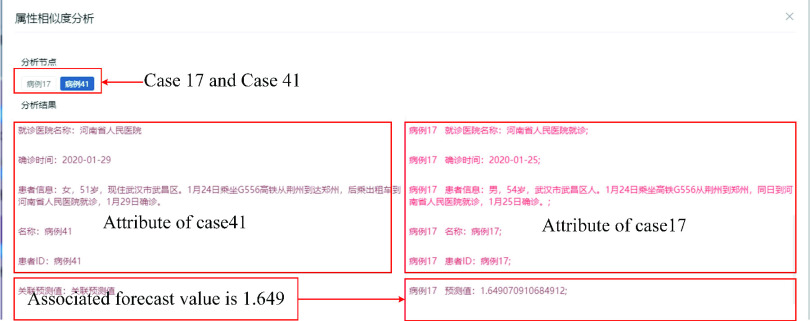
Node attribute similarity calculation.

Node prediction analysis may discover further information. For example, as shown in Figure 11(c), the data show that case 41 and case 758 took the same train on the same day. The similarity analysis of node information is conducted, and a similarity value of 1.5 is obtained. This outcome indicates that case 41, who has not been listed as one of the contactees in the released details, might have contacted case 17. The value of the associated forecast between case 17 and case 41 is 1.649, as shown in Figure 12. Therefore, it is suspected that case 41 (or case 17) is unreported or concealed.

#### 5) Analysis of Patient Trajectories in the City

**FIGURE 13.**
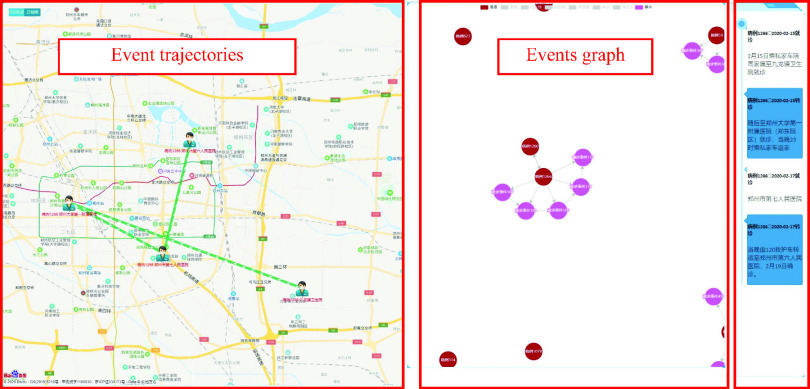
Analysis of patients’ trajectories in urban areas.

The analysis of the patient trajectories in urban areas could help to take particular measures to report and control the places where cases have visited and the public transportation they have taken. In Figure 13, the left graph in the following shows the patient trajectories in the city, the middle graph is the sequential events network of “confirmed diagnosis–confirmed diagnosis–confirmed diagnosis–confirmed diagnosis”, and the right is the event axis.

## VI. Discussion

Experiments have proved that the knowledge graph of the spatiotemporal information of COVID-19 based on various heterogeneous data could take advantage of both semantic web analysis and the visual analysis of the spatiotemporal information for analysis and application to measures that are used to realize “macro situation control” of the epidemic outbreak and “precise prevention and control for patients”. Such measures help to improve the accuracy of locating the epidemic transmission path and preventing the epidemic from further spreading.

However, the database of this article is still relatively small. The proliferation of the data scale and semantic web scale would lead to low analysis efficiency and weak robustness. The knowledge graph of the spatiotemporal information for epidemic prevention and control in the future can be further studied from the following two aspects.

1) The expansion of the data association scale. The data could be improved by adding associated multi-source, cross-domain data types, such as social network data, spatiotemporal location information, epidemic surveillance information and geographic environment data, to build knowledge models and representations suitable for large-scale temporal features. This improvement would help to form a large-scale spatiotemporal information semantic web of the COVID-19 epidemic situation and build up the relationships among epidemic information cross domains and disciplines. For example, the relationship between characters, in addition to “family”, “relatives”, there are “friends”, “colleagues”, “teacher-student” relations, etc. After expanding the character relationship type, a complex character relationship network can be established based on large-scale data such as company human resource data, government household registration data, etc., which would help to identify people suspected of contacting confirmed cases.
2) Use a graph neural network to learn the spatiotemporal knowledge graph. COVID-19 patients’ spatiotemporal information knowledge graph is essentially a large-scale directed graph. A graph neural network model can be used to design a highly dynamic spatial-temporal neural network that meets the sequential characteristics of directed graphs. This design could be used for large-scale node prediction, link prediction and community clustering prediction algorithms in large-scale complex networks to realize the real-time prediction and simulation of the epidemic situation of the COVID-19 spatiotemporal information complex network. For example, we could preset the weights of spatial locations, patients’ influence, hidden relations, and patients’ preferences to study and construct a graph neural network model to implement community detection, epidemic control area classification and analysis of infection patterns.

## VII. Conclusion

In response to the outbreaks, geographers took the initiative to continuously integrate the measures related to space-time big data such as COVID-19 epidemic maps, the distribution of communities with diagnosed residents, and the dynamic trajectory of patients. Their efforts in such areas like the spatial transmission of the epidemic, the dynamic analysis of the disease’s condition and the allocation of emergency resources, might be help of differentiated prevention and control and thus cause social and economic impacts. They have constructed spatial interaction models of population migration and spatial interaction models of epidemics. In general, spatiotemporal big data played a particular role in the emergency stage of the epidemic prevention and control.

In view of the lack of data association in spatiotemporal information analysis and the lack of spatiotemporal situation analysis in knowledge graphs, this article combines the semantic web of the geographic knowledge graph with the visual analysis model of spatial information and puts forward the comprehensive utilization of related technologies of the geographic knowledge graph and big data visual analysis. This realizes the situation analysis of COVID-19 and the exploration of patient relationships through interactive collaborative analysis.

Compared to previously conducted studies, there are seven innovations in this article.

1) It constructs the COVID-19 patients’knowledge graph based on the information of the COVID-19 patients in Henan Province.
2) It conducts patient type analysis, regional prevention, and control situation analysis, gathering infected patients’ analysis, high-risk group prevention and control analysis, city trajectory analysis, and missing report and concealed case analysis to trace the relationships between patients, publish information for high-risk group prevention and control, and analyse event patterns.
3) It designs curve graphs to present the epidemic situation in China and in Henan Province based on the released COVID-19 information that has been analysed using multi-view collaborative analysis technology.
4) It designs a bubble chart showing the development trend of COVID-19 in all provinces and cities in China.
5) It designs a migration chart that is mainly based on the train passenger numbers published on people.com.cn that have reported confirmed cases.
6) It designs a network node chart showing the relationship network in the patient knowledge graph.
7) It designs the distribution map showing the distribution of the daily confirmed cases in Henan Province.

Starting from the analysis of the semantic web and the spatiotemporal distribution, a multi-level analysis and application from “macro situation control” to “precision patient prevention and control” has been implemented, which can be used to improve the scientificity and accuracy of the surveillance, prediction and responses related to new coronavirus pneumonia epidemics. support to further promote the intelligent prevention and control of major epidemic outbreaks and improve the accuracy of locating epidemic transmission path and preventing it from further spreading.
